# Silicon Nitride Bioceramics with TiC Additives: Excellent Mechanical Properties, Cytocompatibility, and Antibacterial Properties

**DOI:** 10.3390/jfb17010020

**Published:** 2025-12-26

**Authors:** Zhebin Lou, Jiayu He, Yuandong Liu, Hanxu Zhu, Xiaofeng Zeng, Zulaikha Abid

**Affiliations:** 1 School of Minerals Processing and Bioengineering, Central South University, Changsha 410083, China; 255611002@csu.edu.cn (Z.L.); 215611005@csu.edu.cn (J.H.); zhuhanxu@csu.edu.cn (H.Z.); 245618003@csu.edu.cn (Z.A.); 2 Key Laboratory of Biohydrometallurgy of Ministry of Education, Changsha 410083, China; 3 Hengyang Kaixin Special Material Technology Co., Ltd., Hengyang 421200, China; zeng_xf8@163.com

**Keywords:** silicon nitride, TiC, mechanical properties, cytocompatibility, antibacterial property

## Abstract

Silicon nitride is a type of bioceramic with great application potential. However, the brittleness of silicon nitride can be addressed through toughening. In this study, various proportions of TiC were incorporated into the sintering additive system to explore the effects of different amounts of TiC on the mechanical properties, cell compatibility, and antibacterial properties of silicon nitride. Silicon nitride was prepared by gas pressure sintering, with TiC addition amounts of 3%, 5%, 8%, and 13% wt. Among the four types of silicon nitride, the mechanical properties of silicon nitride with 3% and 5% wt TiC addition were improved, with the flexural strength and fracture toughness of the former being 571 MPa and 8.35 MPa·m^1/2^, respectively, and the flexural strength and fracture toughness of the latter being 532 MPa and 8.53 MPa·m^1/2^, respectively. The surface of all four types of silicon nitride was enriched with Ti as the amount of TiC added increased, and the surface properties of the four silicon nitrides were the same. All four types of silicon nitride could continuously release Si ions in liquid. *In vitro* cell experiments showed that all four types of silicon nitride could enable normal cell proliferation and adhesion. Silicon nitride with different TiC addition amounts all exhibited good cell compatibility. Compared with the control material, each of the four types of silicon nitride demonstrated robust antibacterial efficacy against *Staphylococcus aureus* and *Escherichia coli*, with comparable potency across all types. These findings indicate that the incorporation of titanium carbide (TiC) within the silicon nitride matrix, particularly within the 3–5% weight ratio range, not only enhances mechanical integrity and cellular compatibility, but also confers notable antibacterial attributes. Consequently, these results demonstrate the promising viability of TiC-modified silicon nitride as a prospective material for the fabrication of bone implants.

## 1. Introduction

The repair capacity of bone tissue for defects is limited. If the damage exceeds the limit of the repair capacity for bone tissue, it is necessary to implant bone tissue engineering materials at the damaged site. As the aging of the population intensifies, the number of patients with bone defects or dysfunction is increasing. Therefore, biocompatibility is a necessary characteristic of bone tissue engineering materials. Biocompatibility is defined as the ability of a material to have an appropriate host response in a specific application [1]. Bone tissue engineering materials are mainly classified into three categories: metallic materials, polymer materials, and bioceramics. Bioceramics are further subdivided into two distinct types: bioactive ceramics and bioinert ceramics. Bioactive ceramics are bioceramics that have the potential to induce specific tissue responses when interacting with the physiological system. Typical types of bioactive ceramics include hydroxyapatite (HA), β-tricalcium phosphate (β-TCP), and bioactive glass [2,3,4]. These materials can promote bone growth and regeneration and have significant effects on repairing damaged or diseased bone tissue. In contrast to bioactive ceramics, bioceramics that do not cause any tissue reactions when interacting with the physiological system are called bioinert ceramics. Common bioactive ceramics include calcium phosphate and bioactive glass, whereas prevalent bioinert ceramics include alumina and zirconia. Bioinert ceramics such as alumina and zirconia have the advantages of high hardness, good biocompatibility, and bonding ability with hard and soft tissues, making them ideal materials for dental applications [5,6,7]. They are widely used in many procedures such as root canal therapy, tooth implantation, and periodontal disease treatment. Nowadays, silicon nitride has entered the researchers’ view as a new type of bioceramic.

Silicon nitride (Si_3_N_4_) is a non-oxide ceramic renowned for its excellent mechanical properties, including high fracture toughness, hardness, and strength [8,9]. Furthermore, silicon nitride ceramics are corrosion-resistant [10] and wear-resistant [11], thus finding wide applications in the fields of machinery and metallurgy. Nonetheless, silicon nitride is susceptible to brittleness, prompting the exploration of various methods to mitigate this drawback. These methods include adding whisker toughening, self-toughening, incorporating second-phase particle toughening, and employing layered composite toughening. Notably, silicon nitride’s most notable attribute lies in biocompatibility [12,13]. Silva et al. [14] conducted *in vitro* cytotoxicity tests using silicon nitride-based ceramics as the research object, and discovered that the culture medium extract of silicon nitride ceramics did not induce cell death, proving the non-toxicity nature of silicon nitride-based ceramics to cells. Fu et al. [15] employed SrO, MgO, and SiO_2_ as sintering aids for silicon nitride materials, respectively. The results of cytotoxicity tests showed that the three types of silicon nitride materials mentioned above dissolved Sr^2+^, Mg^2+^, and Si^4+^ ions in the culture medium, respectively, thereby promoting cell proliferation and differentiation, indicating that these three types of cells had no cytotoxicity. Initially, silicon nitride was considered as a bioinert ceramic. However, Pezzotti proposed that silicon nitride exhibits bioactivity [16,17]. Both *in vitro* cell experiments [18,19,20] and *in vivo* animal experiments [21,22,23,24] have shown that silicon nitride can promote the differentiation of osteoblasts, the formation of new bone, indicating its osteoconductivity. Simultaneously, studies have involved the implantation of silicon nitride into humans and regularly evaluated the silicon nitride implants of patients over long periods. The silicon nitride implants did not cause complications in the human body and maintained good performance over the years [25]. Given that bone tissue engineering materials are implanted into the body, it is crucial to assess their antibacterial qualities. The antibacterial properties exhibited by silicon nitride can reduce the risk of infections caused by implants [26,27,28,29,30]. Silicon nitride has been proven to possess antibacterial effects against *Escherichia coli*, *Staphylococcus epidermidis*, and *Porphyromonas gingivalis* [31]. Additionally, silicon nitride possesses good imaging properties, exhibiting semi-transparency under X-rays, and facilitating observation without obstructing imaging of the bone structures behind the implant. Its excellent dielectric and non-magnetic properties eliminate distortions in CT and MRI scans, preventing the generation of artifacts in CT and MR imaging. This assists doctors in making accurate judgments during surgical procedures, thereby improving treatment outcomes under various medical conditions [32,33].

Titanium carbide (TiC) is a transition metal carbide that has attracted widespread attention due to its remarkable physical and chemical properties. Its characteristics include high hardness, a high melting point, good thermal conductivity, a low thermal expansion coefficient, and excellent oxidation resistance, making titanium carbide play a vital role in various industrial applications [34,35]. Titanium carbide is commonly used to enhance the mechanical properties of metallic and ceramic materials. With its extremely high hardness, titanium carbide is an ideal material for enhancing the hardness of materials. Adding a certain amount of titanium carbide to ceramic materials can significantly improve the hardness and wear resistance of the ceramics [36]. In addition, previous studies have shown that TiC is a good toughening agent for ceramics with high brittleness [37,38]. In the field of biomaterials, TiC is applied as a coating or enhancer, and existing studies have also shown that materials containing TiC coatings or doped with TiC do not affect normal cell proliferation *in vitro* [39,40,41,42,43]. TiC is used as a performance enhancer in the preparation of silicon nitride. TiC-Si_3_N_4_ is a material with excellent physical properties. Wang et al. added TiC to silicon nitride and prepared it using a pressure sintering process, resulting in silicon nitride ceramics with a bending strength of up to 780 MPa and a fracture toughness of up to 8.14 MPa·m^1/2^. Previous studies by Y.L. [44] and W.L. [45] also show that TiC addition effectively improves the flexural strength and fracture toughness of composite materials. Meanwhile, the addition of TiC also enhances the conductivity of silicon nitride ceramics [46]. Previous work by J.Y. [47] has shown that silicon nitride ceramics can self-lubricate by friction when the surface roughness of the contact interface is below a critical threshold. Furthermore, the experimental performance evaluation of full ceramic silicon nitride ball bearings based on the self-lubricating behavior of silicon nitride, as reported by W.P. [48], provides strong validation of this mechanism. Although silicon nitride has self-lubricating properties, the addition of TiC can further enhance the wear resistance of silicon nitride. Shahid et al. found that the addition of TiC increased the hardness of silicon nitride and reduced the friction coefficient and wear rate [49,50]. Investigations by Cai et al. [51] and Saravanan et al. [52] also demonstrated that the incorporation of TiC can significantly enhance the wear resistance of ceramic composites. These findings collectively indicate that TiC contributes to improving the overall reliability of the material, which constitutes the primary motivation for introducing TiC in the present study. Due to the intrinsic self-lubricating behavior of high-hardness silicon nitride materials, the potential adverse effects associated with high hardness—particularly on biological tissues such as bone—can be substantially mitigated in biomedical applications. In comparison, enhancing mechanical strength and fracture toughness to improve material reliability is of greater importance. Therefore, TiC was selected as an additive in the Si_3_N_4_ matrix in this study to achieve improved mechanical performance without compromising its biological applicability. Silicon nitride is a promising biomaterial, so TiC-Si_3_N_4_ also has excellent application potential as a bone transplantation material. The incorporation of TiC into silicon nitride not only enhances mechanical properties such as fracture toughness through a pinning effect but also contributes to the optimization of tribological behavior. Specifically, the introduction of TiC particles mitigates the inherent brittleness of silicon nitride ceramics, leading to a transition in the dominant wear mechanism from pure brittle spalling to a combination of abrasive wear and TiC particle pull-out. This change in wear mechanism can, to some extent, influence and improve the wear resistance of the material. Moreover, the addition of TiC increases the electrical conductivity of silicon nitride bioceramics, thereby opening up the possibility of promoting bone healing through electrical stimulation. This enhanced conductivity also provides a foundation for the development of intelligent biomedical devices, such as active implants capable of monitoring or responding to electrical signals. In addition, the improved electrical conductivity enables the use of efficient fabrication techniques, such as electrical discharge machining, for producing medical components with complex geometries.

This paper will prepare silicon nitride with different titanium carbide additions, aiming to preliminarily demonstrate the feasibility of silicon nitride with TiC as an implant material and explore the effect of TiC addition on the mechanical properties, cell compatibility, and antibacterial properties of silicon nitride. The composition, surface properties, and mechanical properties of silicon nitride with different amounts of titanium carbide were compared. The cytotoxicity of silicon nitride added with different amounts of TiC was detected and compared with ordinary silicon nitride and other control materials. The adhesion and cytoskeleton morphology of cells on the surface of silicon nitride with different amounts of titanium carbide were observed. The antibacterial properties of silicon nitride with different amounts of titanium carbide were tested. This study will provide theoretical support and practical guidance for the optimal design of silicon nitride bioceramics using TiC to enhance performance.

## 2. Materials and Methods

### 2.1. Preparation of Materials

The silicon nitride used in these studies was produced by Hengyang Kaixin Special Materials Technology Co., Ltd. (Hengyang, China). The raw materials used in this study were Si_3_N_4_ powder (D_50_ = 0.65 μm, SBET = 8.7 m^2^/g, Si_3_N_4_ > 99.5%, SN-E10, Ube, Japan), TiC powder (D_50_ = 1.20 μm, chemically pure, Hunan Jingcheng Materials Co., Ltd., Changsha, China) and sintering aids. The original sintering additive system for silicon nitride was Al_2_O_3_, Y_2_O_3_, and MgO, with a total content of 10% wt. The ratio of Al_2_O_3_, Y_2_O_3_, and MgO was 2:5:3. Based on the sintering additive system, TiC was added. The added amounts of TiC were 3%, 5%, 8%, and 13% wt, respectively. The mixture of silicon nitride and the sintering additive powders was added to the milling jar at a ratio of 1:1 to anhydrous ethanol and then milled for 2 h using a planetary ball mill. After the mixed material was completely dried, it was sieved through a 40-mesh screen. The green body was isostatically pressed at 200 MPa and subsequently sintered in a sintering furnace. The preparation of the silicon nitride samples was carried out using gas pressure sintering (GPS) technology, with a sintering temperature set at 1700 °C under a nitrogen pressure of 1.8 MPa. The rate of temperature increase was maintained at 2 °C/min, and the dwell time was set at 2.5 h. After sintering, the surface of the silicon nitride samples was polished using a diamond grinding tool. All the silicon nitride samples were polished using a diamond grinding wheel twice. Firstly, a coarse grinding was performed using 80-mesh diamond particles, and then a fine grinding was carried out using 180-mesh diamond particles. The feed length per time for applied pressure during the fine grinding stage was set as 1 μm, and finally a surface finish of 0.6 μm for all samples was reached. Based on standards, alumina ceramics, titanium alloys, and PEEK were selected as control materials. The control material samples with the same specifications include Alumina (Model: 99%, Foshan Haocai New Material Technology Co., Ltd., Foshan, China), Titanium alloy (Model: CT4, Hebei Bo Titanium Metal Materials Co., Ltd., Langfang, China), and PEEK (Model: Faint yellow, Shenzhen Caocheng Plastic Materials Co., Ltd., Shenzhen, China). All these materials complied with the standards stipulated in GB/T 16886.12 (Biological evaluation of medical devices, Part 12: Sample preparation and reference material, 2005) and ISO 10993-8 (Biological evaluation of medical devices—Part 8: Selection and qualification of reference materials for biological tests, 2000) [53,54]. To meet the requirements of subsequent standardized testing, the materials used in this experiment were all fabricated into discs with a diameter of 10 mm and a thickness of 3 mm.

### 2.2. Physical Characterization of Materials

X-ray diffraction (XRD, RIGAKU, Smartlab SE, Tokyo, Japan) was used to analyze the phase of silicon nitride, and the monochromatic Cu-k α Ray as ray source (λ = 5406 Å), voltage intensity 40 kV, current intensity 40 mA, step size 0.02°, scanning speed 5°/min, scanning range 10–80°. The microstructure of silicon nitride ceramic sheets was observed by scanning electron microscopy (SEM, TESCAN Bmo, s.r.o., TESCAN MIRA, Brno Czech Republic), and the element distribution on the surface of silicon nitride was analyzed by the EDS system. Based on the standards GB/T 6569-2006, GB/T 4740-2024, and GB/T 23806-2009 [55,56,57], the mechanical properties of materials were comprehensively evaluated by an electronic universal testing machine (MTS, CMT6103, Eden Prairie, MN, USA), including bending strength, compressive strength and fracture toughness. The bending strength was determined using the three-point bending method, with a span size of 30 mm and a loading speed of 0.5 mm/min. The sample size used for testing was 3 mm × 4 mm × 36 mm. When testing compressive strength, apply a loading rate of 3 mm/min until the interface of the material is damaged, and record the maximum load. The fracture toughness was determined using the single-sided notched beam method (SENB) with a sample size of 3 mm × 4 mm × 36 mm. Cracks were introduced in the middle of the sample through manual slotting, with a depth of 2 mm and a width of 0.2 mm. Test conditions: span size of 25 mm, loading speed of 0.05 mm/min. Measure the Vickers hardness of the sample with a hardness tester (INNOVATEST FALCON507, Maastricht, The Netherlands), set the indenter of the hardness tester to apply a pressure of 98.1 N (10 kg) to the silicon nitride material for about 15 s, and calculate the Vickers hardness value of the material according to the size of the dent left by the indenter on the sample surface. The wettability of the material was measured using a contact angle tester (Chengde Dingsheng, JY-82C, Chengde, China), and the water contact angle was measured by dropping a small amount of water on the surface of the material using the seat drop method. By using a three-dimensional laser micro imaging system (KEYENCE, VK-X150, Osaka, Japan), the contour and texture of the sample material surface can be accurately captured at the micro level to measure the surface roughness of the material. The silicon nitride samples were immersed in simulated body fluid (SBF, Phygene, Fuzhou, China) in a ratio of 1.25 cm^2^/mL of sample surface area to solution volume for 3, 7, and 14 days. The dissolved Si ion concentrations of the samples at different time points in the SBF solution were analyzed using ICP-OES (Agilent, 720ES (OES), Santa Clara, CA, USA). After immersing for 3 to 7 days, the dissolved Ti and Y ion concentrations of samples at different time points in SBF solution were measured.

### 2.3. Cytotoxicity Test

The mouse embryonic osteoblast precursor cells (MC3T3-E1, Procell Life Science & Technology Co., Ltd., Wuhan, China), commonly used as a skeletal research model cell line, were employed in this *in vitro* cellular experiment. The MC3T3-E1 cells were cultured in a complete culture medium (Procell Life Science & Technology Co., Ltd., Wuhan, China), which was based on α-MEM as the base medium and supplemented with 1% (*v*/*v*) penicillin–streptomycin solution and 10% (*v*/*v*) fetal bovine serum. A carbon dioxide incubator (Zhicheng Inc., Shanghai, China) was used to maintain the cells at 37 °C in a 5% CO_2_ environment to sustain their physiological activity and carry out subsequent experimental operations. Passage of cells was performed when the cell coverage in the culture dish reached 70–90%. After 3–5 passages, the cells were seeded onto ceramic disks.

The collected cells were resuspended at 3 × 10^4^ cells/mL in complete medium. The sterilized material was placed in a sterile 24-well cell culture plate (Saining Biotechnology Co., Ltd., Suzhou, China), and the cell suspension was added dropwise. Put the well plates into the cell culture incubator for 1, 3, and 5 days. The culture medium was changed every 2–3 days. On the 1st, 3rd and 5th day, the original culture medium was removed, and the cells were gently rinsed with PBS two to three times. The medium containing 10% CCK-8 solution (Beyotime Biotech, Shanghai, China) was added and incubated in the incubator for 2 h. Its absorbance at 450 nm was measured with a microplate reader (BioTek instruments. Inc., epoch™, Winooski, VT, USA).

### 2.4. Cell Adhesion and Morphology

Put the sample material into a 24-well cell culture plate, dilute MC3T3-E1 cells to 4 × 10^4^ cells/mL, then add it to the well plate, and put it into a carbon dioxide incubator for 24 h. After 24 h, the original culture medium was removed, gently washed with PBS two to three times, and the cells were fixed by adding 4% paraformaldehyde fixation solution at a low temperature overnight. DAPI (Beyotime Biotech, Shanghai, China) and actin tracker red rhodamine (Beyotime Biotech, Shanghai, China) were used for staining, and the cytoskeleton morphology was observed by confocal laser scanning microscope (CLSM, Zessi, lsm800, Oberkochen, Germany).

Put the sample material into a 24-well cell culture plate, dilute MC3T3-E1 cells to 4 × 10^4^ cells/mL, then add it to the well plate, and put it into a carbon dioxide incubator for 24 h. After 24 h, the original culture medium was removed, gently washed with PBS two to three times, and 2.5% glutaraldehyde fixative was added to fix the cells on the sample at low temperature overnight. The samples were dehydrated in a graded ethanol solution (30, 50, 70, 85, 90, and 100%, respectively) for 10 min and dried at the critical point. The morphology of adherent cells on the materials was observed by scanning electron microscopy (Hitachi, Regulus8100, Tokyo, Japan).

### 2.5. Evaluation of Antibacterial Property

*Staphylococcus aureus* (ATCC 25923) and *Escherichia coli* (ATCC 25922), as common Gram-positive and Gram-negative bacteria, were used to evaluate the antibacterial properties of the materials. Using the colony counting method, the number of bacteria in the bacterial solution was estimated by calculating the number of colonies on the agar plate.

The sample material was put into a 24-well cell culture plate after high-temperature sterilization, and an equal amount of bacterial solution was added dropwise onto the material’s surface, and placed in a constant temperature and humidity incubator for 24 h at 37 °C. After that, the material was cleaned with PBS, and the eluent was serially diluted ten times gradient to the appropriate concentration, and an appropriate volume of final diluent was then dropped onto an agar plate. The bacterial solution was evenly spread on the plate, which was then cultured in a constant temperature and humidity incubator at 37 °C until the plate appears colonies. Finally, the number of colonies on each plate was counted.

### 2.6. Statistical Analysis

Each experiment used three experimental samples for statistical analysis of experimental data. The mean ± standard deviation was used to represent the experimental results. Use statistical product and service solution 26.0 software (SPSS 26.0, IBM, New York, NY, USA) to conduct student *t*-tests and one-way analysis of variance (ANOVA) on the data * *p* < 0.05 indicated statistical significance.

## 3. Results

### 3.1. Characterization of Materials

#### 3.1.1. SEM Observation of Materials

**Figure 1:**
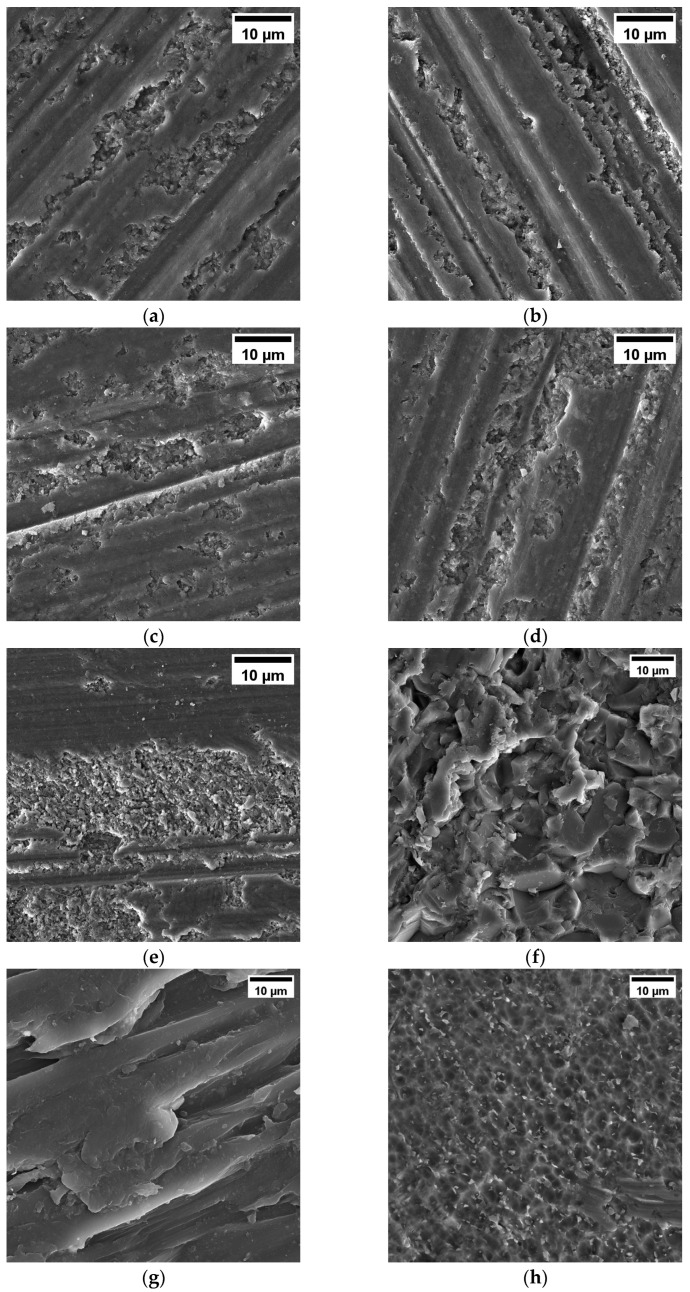
SEM image of surface morphology of silicon nitrides and control materials (magnification factor: 4.00 k×). (**a**) SN + 3% wt TiC, (**b**) SN + 5% wt TiC, (**c**) SN + 8% wt TiC, (**d**) SN + 13% wt TiC, (**e**) SN, (**f**) Alumina ceramic, (**g**) Titanium alloys, (**h**) PEEK.

The SEM results of the sample surface morphology are shown in Figure 1, in which a–e successively show the surface morphology of silicon nitride with titanium carbide addition content of 3%, 5%, 8%, and 13% wt and without TiC, and f–h successively show the surface morphology of aluminum oxide, titanium alloy, and PEEK. Silicon nitride with TiC as an additive was prepared via an air pressure sintering process, followed by surface polishing with a diamond grinding wheel, forming in a unique surface morphology. The surface morphology of silicon nitride samples with four different TiC contents is very similar to that of the original silicon nitride without TiC. Significant stripe lines can be observed in the corresponding SEM images. These stripes originate from the surface being ground by the diamond grinding wheel, and the stripes show non-uniformity in width and depth on the micro scale. A large number of pits with different sizes along with uneven distribution can be observed inside and around the stripes. Obvious silicon nitride crystal particles can also be observed from the pits. As a control material, irregular holes and alumina crystals of varying sizes are distributed on the surface of alumina. There are dense crystals on the surface of the titanium alloy. On the surface of PEEK, protrusions of different lengths are distributed, and there are gullies of different widths between the protrusions.

**Figure 2:**
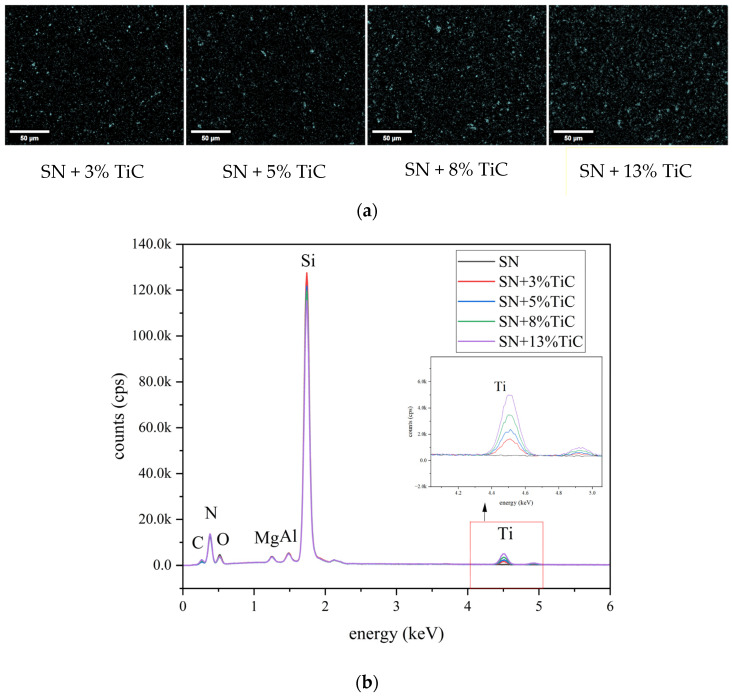
EDS analysis of surface elements of silicon nitrides containing TiC. (**a**) Distribution of Ti elements on the surface of silicon nitrides with different TiC content. (**b**) Surface element atlas of silicon nitrides with different TiC content.

SEM was used to observe the surface morphology of silicon nitride with four different TiC additions, and the EDS system was used to analyze the element on the material surface. The EDS analysis of surface elements of silicon nitride with different TiC addition amounts is shown in Figure 2. Figure 2a shows the Ti element distribution of silicon nitride samples with 3%, 5%, 8% and 13% TiC content. Figure 2b and Table 1 present the energy spectrum curve of the corresponding detection site. According to Figure 2a, the distribution of Si, N and Ti on the surface of silicon nitride with four types of TiC content is observable. The presence of Si, N, and Ti can be observed on the surface of the four types of silicon nitride. The density of Si represented by the color purple, is the highest in the figure, indicating the highest Si content. The density of N is weaker compared to that of Si, while the density of Ti is the lowest. The distribution of Si and N is consistent with the surface morphology of silicon nitride, where the stripes and pits on the surface of silicon nitride are also visible in the distribution of Si and N. The distribution of Ti is different. The distribution of Ti is relatively dispersed and concentrated in the form of specific location points. With the increase in the amount of TiC added, the distribution of Ti becomes denser and obvious. The sample spectrum in Figure 2b and Table 1 reveals the presence of Si, N, Al, Ti, Mg and other elements on the surface of silicon nitride. Al and Mg are derived from Al_2_O_3_ and MgO, the sintering additives of silicon nitride. The Si peak is located at 1.74 keV, and its intensity is the highest. This is consistent with the results in Figure 2a. With the increase in TiC content, the intensity of the Si peak decreases. The Ti peak is at 4.51 keV. As shown in the inset of Figure 2b, its intensity increases with the increase in TiC addition, while no Ti peak is detected in silicon nitride without TiC addition.

#### 3.1.2. XRD of Silicon Nitride with TiC

**Figure 3:**
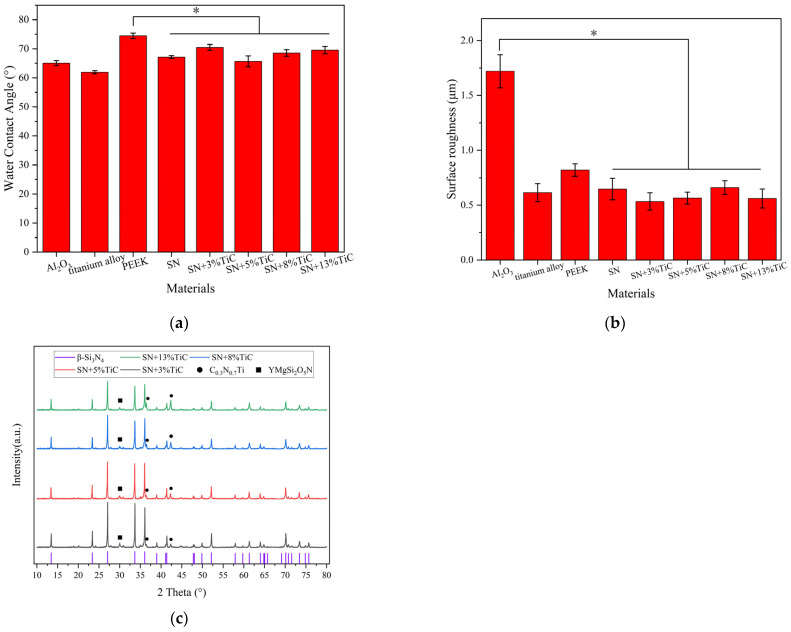
(**a**) Wettability of each material, (**b**) Surface roughness of each material, (* *p* < 0.05) (**c**) XRD of silicon nitride samples.

Figure 3a presents the XRD patterns of various silicon nitride materials. It can be observed that silicon nitride with different concentrations of titanium carbide addition all exhibit the main phase of β-Si_3_N_4_. The XRD spectra of these four silicon nitride materials all show characteristic peaks of β-Si_3_N_4_ (indicated by purple solid line) at 2θ = 13.1°, 22.8°, 27.3°, 33.2°, 36.1°, 41.2°, 52.3°, and 70.1°. As the amount of TiC added increases, the intensity of the β-Si_3_N_4_ characteristic peaks at 27.1°, 33.7°, and 36.1° decreases, which is consistent with the decrease in silicon nitride content as the TiC content increases. In addition, the spectra also show characteristic peaks of two other substances, namely C_0.3_N_0.7_Ti and YMgSi_2_O_5_N. The characteristic peaks of C_0.3_N_0.7_Ti are located at 2θ = 36.7° and 42.4° (marked with black solid circles), and the intensity of these peaks increases with the increase in TiC content. YMgSi_2_O_5_N mainly exhibits a characteristic peak at 2θ = 30.1° (marked with black solid squares) in the spectra, and its peak intensity is inversely related to that of C_0.3_N_0.7_Ti. The intensity of the YMgSi_2_O_5_N characteristic peak decreases as the amount of TiC added increases.

#### 3.1.3. Surface Roughness and Wettability of Materials

The wettability test results for various sample materials are presented in Figure 3b, revealing the water contact angles of different materials. It is evident that silicon nitride with 3% titanium carbide addition exhibits the largest water contact angle, measuring 76.49 ± 0.99°, followed by PEEK with a contact angle of 74.46 ± 0.34°. The water contact angles of alumina and silicon nitride are relatively similar, with values of 64.92 ± 0.53° and 65.53 ± 0.21°, respectively. Titanium alloy has the smallest water contact angle, at 61.94 ± 0.56°. It can be observed that the addition of different concentrations of titanium carbide to silicon nitride results in an increase in its water contact angle, with values of 76.49 ± 0.99°, 65.65 ± 0.31°, 68.57 ± 0.62°, and 71.56 ± 0.28° in ascending order.

The surface roughness of four types of silicon nitride with different TiC additions was examined. The results are presented in Figure 3c. The surface roughness for silicon nitride with 3%, 5%, 8%, and 13% TiC additions were 0.53 ± 0.08 μm, 0.56 ± 0.05 μm, 0.66 ± 0.11 μm, and 0.56 ± 0.09 μm, respectively. The surface roughness of silicon nitride without TiC was 0.64 ± 0.09 μm. Therefore, the surface roughness of silicon nitride treated with the same diamond grinding wheel falls within a range of 0.5–0.6 μm. For comparison, the surface roughness values of alumina, titanium alloy, and PEEK were1.72 ± 0.15 μm, 0.61 ± 0.08 μm, and 0.82 ± 0.06 μm, respectively. It can be observed that the surface roughness of silicon nitride with 3%, 5%, and 13% TiC content is lower than that of silicon nitride without TiC, titanium alloy, and PEEK.

#### 3.1.4. Si, Ti and Y Ions Dissolution Determination

**Figure 4:**
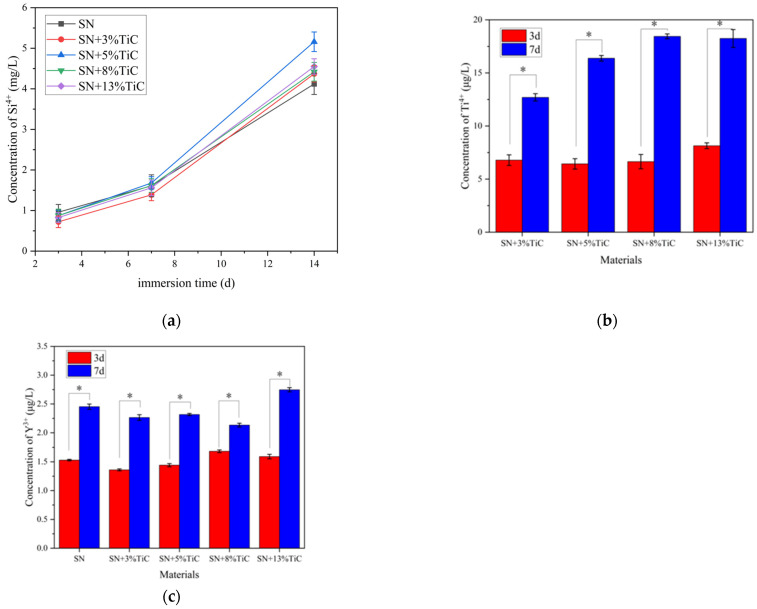
Release of Si ions (**a**), Ti ions (**b**), and Y ions (**c**) from silicon nitride with added TiC in SBF (* *p* < 0.05).

When four types of silicon nitride with different TiC contents were immersed in SBF and compared with silicon nitride without TiC, the release of Si ions was measured at different time points. The results are presented in Figure 4a. The four silicon nitride samples were immersed in SBF for 3, 7, and 14 days, respectively. During this period, all four silicon nitride samples continuously released Si ions. The concentration of Si ions in the SBF extracts of the four silicon nitride samples continued to increase, and on the 14th day, the Si ions concentration was higher than that of silicon nitride without TiC. On the 14th day, the silicon nitride with 5% wt TiC content had the highest Si ions concentration level, reaching 5.16 mg/L. The Si ions concentration levels of silicon nitride with 3%, 8%, and 13% wt TiC contents were relatively close, basically at the same level, with Si ions concentrations recorded as 4.37, 4.42, and 4.55 mg/L, respectively. The concentrations of Ti ions and Y ions in the silicon nitride extraction solution are shown in Figure 4b and Figure 4c, respectively. The concentrations of Ti ions and Y ions in each silicon nitride extraction solution increased with time. Although the release concentration of Si ions was as high as mg/L during the same immersed time, the concentration of these two metal ions released by silicon nitride was significantly lower, only at the level of μg/L, forming a sharp contrast. This indicates that the release rate of Ti ions and Y ions in silicon nitride is much lower than that of Si ions. There was also a difference between the concentration of Ti ions and Y ions, with Ti ions having a higher concentration than Y ions. After immersing for 3 days, the Ti ions concentration in the extraction solution of silicon nitride with different TiC addition amounts was higher than 6 μg/L. The concentration range of Y ions was 1.3–1.6 μg/L, and the highest concentration of silicon nitride extraction solution with 8% wt TiC added was 1.68 ± 0.02 μg/L. After immersing for 7 days, as the TiC content increased, the Ti ions concentration increased. The Ti ions concentration in the silicon nitride extract with 13% wt TiC added was as high as 18.24 ± 0.84 μg/L, and the Ti ions concentration in the silicon nitride extract with 3% wt TiC added was 12.69 ± 0.34 μg/L. The concentration range of Y ions in silicon nitride extraction solutions with different TiC addition amounts ranges from 2.1 to 2.7 μg/L, and the highest concentration of silicon nitride extraction solution with 13% wt TiC addition is 1.68 ± 0.02 μg/L.

### 3.2. Mechanical Properties of the Materials

Using an electronic universal testing machine, the mechanical properties of silicon nitride with TiC additions of 3%, 5%, 8%, and 13% wt (SN + 3% TiC, SN + 5% TiC, SN + 8% TiC, SN + 13% TiC) were measured and compared with those of silicon nitride without TiC (SN), alumina, PEEK, and titanium alloy. The results of the mechanical properties of each material are summarized in Table 2. The Vickers hardness of silicon nitride with different TiC contents was also examined. The results indicate that the Vickers hardness of silicon nitride exhibits a trend of initially increasing and then decreasing as the amount of TiC addition increases. Silicon nitride with 3% and 5% TiC additions exhibited higher Vickers hardness values compared to the original silicon nitride without TiC, with values of 15.02 ± 0.28 GPa and 15.23 ± 0.33 GPa, respectively. Notably, the silicon nitride with 5% TiC addition exhibited the highest Vickers hardness among them. Subsequently, as the TiC addition increased to 8% and 13%, the Vickers hardness of the corresponding silicon nitride samples decreased to 14.51 ± 0.31 GPa and 14.13 ± 0.39 GPa, respectively. Both of these values are lower than that of the original silicon nitride without TiC addition. Silicon nitride samples with different TiC additions exhibited Vickers hardness values that were all higher than those of titanium alloys, being approximately four times harder. In terms of flexural strength, it initially increased and then decreased with the addition of TiC. Silicon nitride with 3% TiC addition demonstrated the highest flexural strength, reaching 571 ± 29.3 MPa, which surpassed that of the original silicon nitride without TiC. As the TiC content increased, the flexural strength of silicon nitride samples with 5%, 8%, and 13% TiC additions gradually decreased to 532 ± 30.9, 482 ± 32.7, and 435 ± 31.0 MPa, respectively. Silicon nitride samples with 8% and 13% TiC additions exhibited lower flexural strength than the original silicon nitride. Notably, all the silicon nitride samples exhibited superior flexural strength compared to alumina and PEEK. Regarding compressive strength, a similar trend was observed. The compressive strength initially increased and then decreased with the addition of TiC. Silicon nitride with 3% TiC addition exhibited the highest compressive strength, reaching 2537 ± 132.9 MPa, which was also higher than the original silicon nitride. The compressive strength of silicon nitride samples with 5%, 8%, and 13% TiC additions gradually decreased to 2431 ± 133.9, 2315 ± 138.4, and 2114 ± 131.9 MPa, respectively. Silicon nitride samples with 5%, 8%, and 13% TiC additions exhibited lower compressive strength than the original silicon nitride. Notably, the compressive strength of all four silicon nitride samples was approximately three times higher than that of titanium alloys and 20 times higher than that of both PEEK and human bone, being comparable to the compressive strength of alumina. In terms of fracture toughness, this parameter increased with the addition of TiC. Silicon nitride samples with 3% and 5% TiC additions exhibited higher fracture toughness than the original silicon nitride without TiC, with values of 8.35 ± 0.622 MPa·m^1/2^ and 8.53 ± 0.663 MPa·m^1/2^, respectively. Among them, the sample with 5% TiC addition demonstrated the highest fracture toughness. Up to 5% TiC addition, TiC effectively enhanced the toughness of the material. However, as the amount of TiC added further increased, the fracture toughness began to decrease. Silicon nitride samples with 8% and 13% TiC additions exhibited fracture toughness values of 7.66 ± 0.642 MPa·m^1/2^ and 6.55 ± 0.601 MPa·m^1/2^, respectively. Notably, only the sample with 13% TiC addition exhibited a lower fracture toughness than the original silicon nitride. Although the fracture toughness of these silicon nitride samples was approximately one-tenth of that of titanium alloys, they still outperformed alumina.

**Figure 5:**
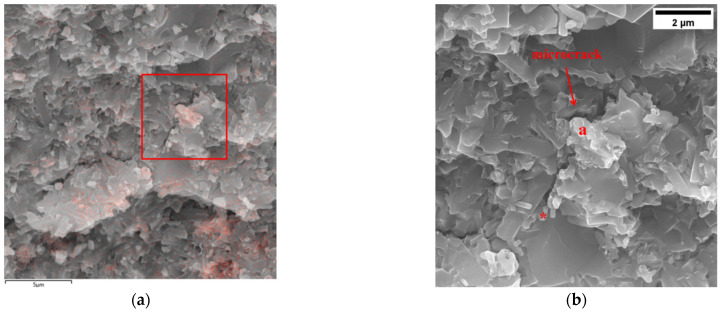
5% wt TiC addition silicon nitride cross-section ((**a**) EDS + SEM, 10 k×, (**b**) SEM, 20 k×). * The microcrack starts to grow upwards from the bottom.

The cross-section of silicon nitride containing TiC was observed using SEM, as shown in Figure 5. Figure 5a combines the EDS results, where the red spot represents the Ti element. Ti is concentrated at specific locations in the cross-section of silicon nitride, which is consistent with the results in Figure 2. Figure 5b is a partial enlargement of the red box position in Figure 5a. It can be clearly seen that microcrack starts to grow upwards from the bottom *, and when passing through the Ti enriched point a, the crack deflects and continues to grow.

As shown in Figure 5a, when the TiC content is excessively high, TiC particles tend to preferentially segregate along the Si_3_N_4_ grain boundaries rather than being uniformly dispersed within the matrix. This grain boundary segregation disrupts the continuity of the intergranular phase in Si_3_N_4_ and weakens the grain boundary bonding. Furthermore, the significant mismatch in elastic modulus and coefficient of thermal expansion between TiC and the Si_3_N_4_ matrix leads to localized stress concentrations during sintering and subsequent mechanical loading. From Figure 5b, it can be observed that microcracks form in the TiC-rich grain boundary regions, indicating that these interfaces act as preferential sites for crack nucleation. Under bending or compressive loads, these microcracks readily propagate along the weakened grain boundaries, thereby accelerating fracture and resulting in reduced bending strength and compressive strength. This effect becomes increasingly pronounced at higher TiC contents (8–13 wt%), where excessive second-phase aggregation outweighs the potential toughening benefits that TiC could otherwise provide. In contrast, at lower TiC additions, TiC particles are more uniformly distributed and can effectively hinder crack propagation without severely compromising grain boundary integrity, thereby maintaining or even enhancing the mechanical properties. Together, these observations account for the decline in strength observed at elevated TiC contents.

### 3.3. Evaluation of Cytotoxicity of the Silicon Nitrides with TiC

**Figure 6:**
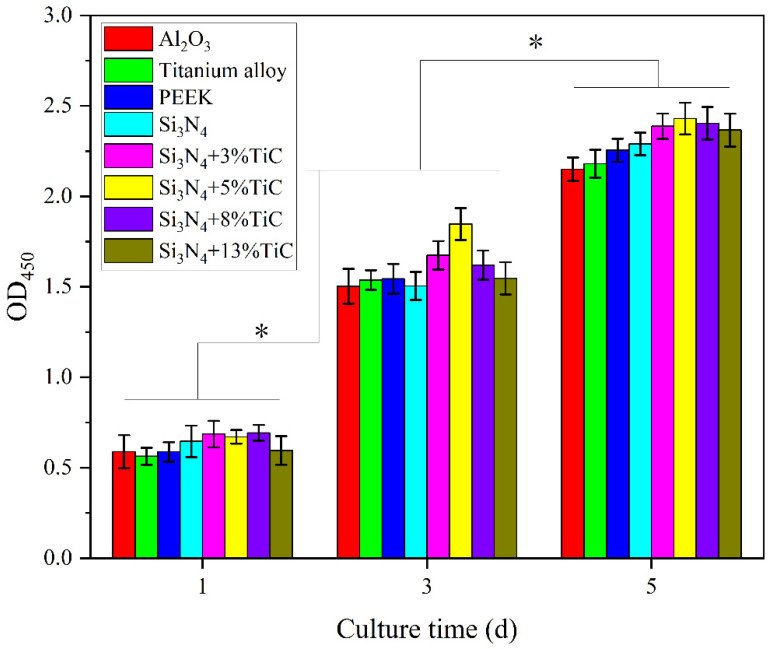
OD value of MC3T3-E1 cells culturing on the samples for different times. (* *p* < 0.05).

MC3T3-E1 cells were co-cultured with four types of silicon nitride with different TiC content, silicon nitride without TiC and MC3T3-E1 cells for 1, 3 and 5 days, respectively. The cytotoxicity of the above five kinds of silicon nitride was mainly detected by CCK-8 method because it provides a quantitative and reproducible measurement of cell viability by detecting the OD_450_ value, and can also reflect the proliferation of MC3T3-E1 cells at different culture times. The results are shown in Figure 6. OD_450_ reflects the number of cells, where a higher OD_450_ indicates more cells. After one day of culture, the OD_450_ of silicon nitride with four different TiC contents was higher than that of the control material and was equivalent to that of silicon nitride without TiC. The OD_450_ of silicon nitride with 13% wt TiC content was lower than that of the other three kinds of silicon nitride with TiC, but there was no significant difference between the five types of silicon nitride. With the increase in time, the OD_450_ corresponding to each material increased significantly. This indicates that the cells can maintain normal physiological activities and proliferate when co-cultured with various materials. On the third day, the OD_450_ of all materials increased significantly, and the OD_450_ corresponding to 5% wt TiC content silicon nitride was the highest. On the fifth day, the OD_450_ of all silicon nitride samples was higher than that of the control material, and the OD_450_ of silicon nitride containing TiC additive was higher than that of silicon nitride without TiC, but there was no significant difference among the five silicon nitrides. This indicates that the addition of TiC will not cause cytotoxicity to silicon nitride, and silicon nitride will also not produce cytotoxicity with the increase in TiC addition.

### 3.4. Cell Adhesion and Morphology of the Silicon Nitrides with TiC

**Figure 7:**
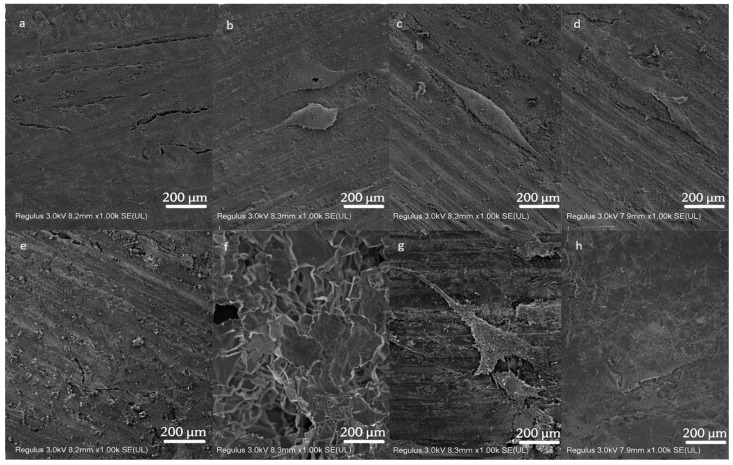
SEM images of MC3T3-E1 cell morphology on the silicon nitrides and control materials (magnification factor: 1.00 k×). (**a**) SN + 3% wt TiC, (**b**) SN + 5% wt TiC, (**c**) SN + 8% wt TiC, (**d**) SN + 13% wt TiC, (**e**) SN, (**f**) Alumina ceramic, (**g**) Titanium alloys, (**h**) PEEK.

SEM observations are mainly used to evaluate cell attachment, spreading behavior, and cell–material interactions at the early stage. The adhesion of cells on silicon nitride with different amounts of TiC is shown in Figure 7. It can be observed that there is cell adhesion on the surface of all materials. The adhesion of cells covers the special texture and morphology of the surface of various materials, especially silicon nitride. The morphology and location of cell adhesion depend on the surface morphology of the material. Multiple cells can be observed on the surface of five kinds of silicon nitride. The cells adhere to the surface of silicon nitride in a flat sheet or spindle shape. The sheet-like cells are divergent and extend many pseudopodia around. The spindle cells mainly extend pseudopodia at the thinner ends to complete cell adhesion. On the surface of silicon nitride, there are strip lines and irregular pits exposing silicon nitride crystals due to grinding. These structures provide adhesion sites for cell pseudopodia. In Figure 7a,e, the cells are flat and cover the surface of the material to block the surface texture of the material. In Figure 7a, it can be observed that multiple sheets of cells are connected to each other, but the specific cell boundaries of interconnected cells cannot be distinguished. The cells in Figure 7b,c appear fuller compared to those in Figure 7a,e, which is likely due to the differences in cell adhesion and spreading time. The longer the adhesion time, the more prone the cells are to change from a plump and protruding state to a flat and flaky state. In Figure 7b–d, the direction of cell adhesion is consistent with the surface texture of silicon nitride. According to Figure 7a–e, the addition of TiC and the increase in the amount of TiC did not affect the cell adhesion on the corresponding silicon nitride surface. The adhesion morphology of cells on the surface of titanium alloy and PEEK showed irregular sheets and extended pseudopodia connecting with each other. The adhesion of cells on the surface of alumina ceramics is slightly different from that of other materials. According to the surface morphology of alumina ceramic in Figure 1, many alumina crystals are distributed on its surface, providing adhesion sites for cells. In Figure 7f, cells adhere to the surface of silicon nitride by wrapping around prominent alumina crystals. The absence of an obvious proliferation difference in Figure 7 does not contradict the quantitative viability results. Figure 7 confirms that the addition of TiC does not impair cell adhesion or morphology, while quantitative proliferation and viability are appropriately reflected by the CCK-8 results in Figure 6.

**Figure 8:**
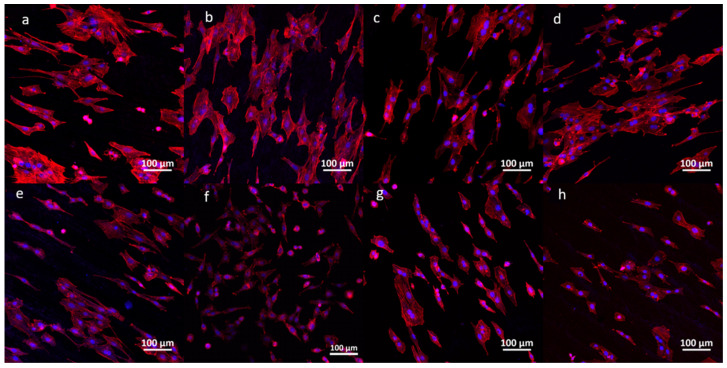
CLSM images of MC3T3-E1 cell cytoskeleton morphology on the silicon nitrides and control materials. (**a**) SN + 3% wt TiC, (**b**) SN + 5% wt TiC, (**c**) SN + 8% wt TiC, (**d**) SN + 13% wt TiC, (**e**) SN, (**f**) Alumina ceramic, (**g**) Titanium alloys, (**h**) PEEK.

The cytoskeleton morphology on silicon nitride and control materials with different TiC addition amounts is shown in Figure 8. The nucleus combines with DAPI dye to emit blue fluorescence, and actin in microfilaments combines with microfilament red fluorescent probe to emit red fluorescence. It can be observed from Figure 8 that the cytoskeleton morphology of cells on silicon nitride with different TiC content differs from the three control materials, especially alumina and PEEK. The cells are densely distributed in silicon nitride with different TiC content and silicon nitride without TiC, and the cells are divergent or spindle-shaped. At the same time, multiple cells are connected through the cytoskeleton to form a sheet structure. A large number of actin microfilaments form the cytoskeleton in each cell. It can be observed in the figure that the fluorescence intensity of the pseudopodia protruding from the cell is stronger, indicating that the density of the microfilament skeleton formed by the protruding pseudopodia actin is higher. Different from the skeleton of cells on silicon nitride, although the number of cells on the surface of alumina is also large, the cells basically adhere to the surface of the material in a single independent form, and there is no connection between cells. The reason for this phenomenon is that there are alumina crystals on the surface of alumina, and the material surface is not smooth and continuous. As shown in Figure 1, the material surface is not smooth and continuous, resulting in a single cell adhering to the crystals. There is less cross-linking with PEEK cells, and the cells are basically in a single adhesion state, which is related to the surface morphology shown in Figure 1.

### 3.5. Evaluation of Antibacterial Property of the Silicon Nitrides with TiC

**Figure 9:**
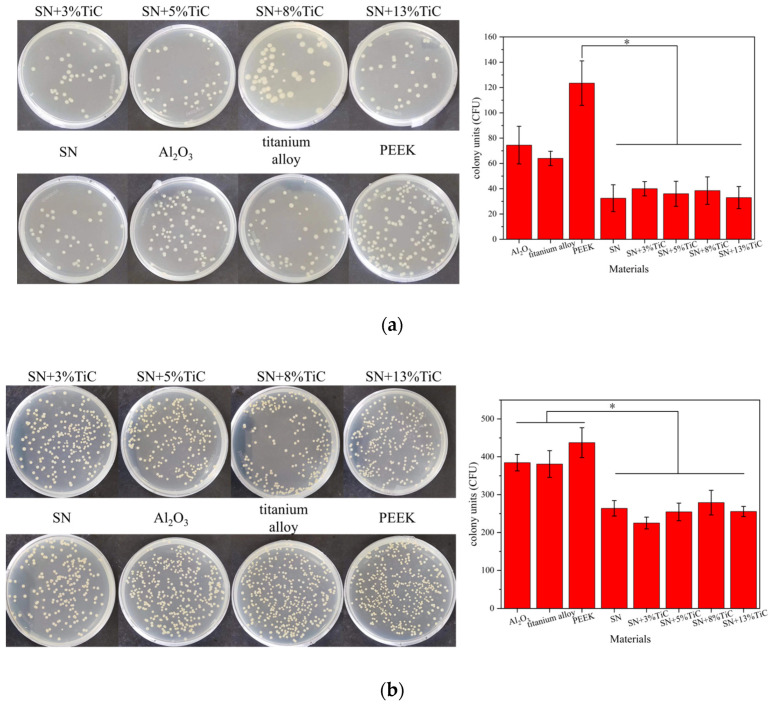
Antibacterial properties of different samples. (**a**) Photographs of *E. coli* colonies on the agar plates, and comparison of the number of CFUs on different materials of *E. coli*, (**b**) Photographs of *S. aureus* colonies on the agar plates, and comparison of the number of CFUs on different materials of *S. aureus*. (* *p* < 0.05).

The antibacterial properties of silicon nitride with different TiC additions against *E. coli* and *S. aureus* were evaluated as shown in Figure 9. Figure 9a displays the colony plates of *E. coli* cultured on agar plates after inoculation and elution on the materials, while Figure 9b shows the colony plates of *S. aureus* cultured under similar conditions. Figure 9a reveals that the number of colonies on the plates corresponding to silicon nitride with different TiC contents is lower than that of the three control materials. The colony counts of *E. coli* on the plates for SN, SN + 3% TiC, SN + 5% TiC, SN + 8% TiC, and SN + 13% TiC are 32 ± 10.6, 40 ± 5.7, 36 ± 9.9, 38 ± 10.8, and 33 ± 8.7, respectively. In contrast, the colony counts of *E. coli* on the plates for alumina, titanium alloy, and PEEK are 74 ± 14.8, 64 ± 5.7, and 123 ± 17.6, respectively. There are no significant differences in the number of colonies among the *E. coli* plates corresponding to silicon nitride with different TiC additions. Therefore, it is believed that TiC does not have a significant impact on the antibacterial effect of silicon nitride against *E. coli*. The antibacterial effect of silicon nitride against *S. aureus* is similar to that against *E. coli*. Figure 9b demonstrates that the colony counts on the plates corresponding to three types of silicon nitride are also lower than those of the three control materials. The colony counts of *S. aureus* on the plates for SN, SN + 3% TiC, SN + 5% TiC, SN + 8% TiC, and SN + 13% TiC are 264 ± 20.4, 225 ± 15.5, 254 ± 23.3, 279 ± 32.5, and 255 ± 13.5, respectively. In contrast, the colony counts of *S. aureus* on the plates for alumina, titanium alloy, and PEEK are 384 ± 21.8, 381 ± 35.4, and 437 ± 39.5, respectively. Similarly, there are no significant differences in the number of colonies among the *S. aureus* plates corresponding to silicon nitride with different TiC additions. Therefore, it is concluded that TiC does not significantly affect the antibacterial effect of silicon nitride against *S. aureus*.

## 4. Discussion

In this study, silicon nitride with different TiC additions were introduced. In terms of material characterization, the mechanical properties, phase state, surface element distribution, surface roughness, and wettability of silicon nitride with different TiC contents were compared. Regarding biological performance, the cytotoxicity, cell adhesion, and cytoskeleton morphology of silicon nitride with different TiC contents were compared. The resistance to microorganisms is also an important performance of bioceramics. Therefore, the resistance of silicon nitride with different TiC addition amounts to *S. aureus* and *E. coli* has also been evaluated.

Silicon nitride was prepared using gas sintering process, and the sintering aid system used was Al_2_O_3_-MgO-Y_2_O_3_-TiC, with TiC addition amounts of 3%, 5%, 8%, and 13% wt, respectively. The XRD results indicate that the main phase of the silicon nitride sample is β-Si_3_N_4_. After mixing α-Si_3_N_4_ raw material with sintering aids, at high temperatures α-Si_3_N_4_ converts to β-Si_3_N_4_. Additionally, C_0.3_N_0.7_Ti and YMgSi_2_O_5_N are present in the sample. YMgSi_2_O_5_N is the main crystalline phase of oxygen–nitrogen microcrystalline glass formed during the sintering process of silicon nitride. During the high-temperature sintering process, the nitridation of TiC_x_ derived from the dissolution of C and N atoms leads to the formation of a new phase C_0.3_N_0.7_Ti [59,60]. Due to the strong covalent bonding of silicon nitride, pure silicon nitride powder is difficult to densify during high-temperature sintering, which requires the addition of sintering aids to resolve this problem. Consequently, metal oxides such as MgO and rare-earth oxides such as Y_2_O_3_ are commonly introduced as sintering additives during the fabrication of Si_3_N_4_ ceramics. During high-temperature sintering, the melting of these additives leads to the formation of a transient liquid phase, which progressively wets the initial grain boundaries. As a result, the grain boundaries transform from solid–solid interfaces into solid–liquid interfaces, thereby significantly promoting particle rearrangement, diffusion, and interparticle contact, as well as facilitating the α-to-β phase transformation of Si_3_N_4_. This process promotes the growth of elongated β-Si_3_N_4_ grains, and the resulting interlocking microstructure is beneficial for crack deflection and crack-bridging mechanisms, leading to enhanced fracture resistance and mechanical reliability. In addition, the formation of Y–Si–Mg–O–N glassy or partially crystallized grain boundary phases contributes to grain boundary stabilization and improved intergranular bonding. During the cooling stage, these sintering additives may precipitate at the grain boundaries, forming secondary phases that further strengthen grain boundary cohesion and influence the overall mechanical performance of the ceramic. Moreover, magnesium is a biologically relevant element associated with bone metabolism; therefore, the presence of MgO as a sintering aid may exert a positive effect on cellular responses.

TiC was used as a reinforcement agent to be added to silicon nitride, while Y_2_O_3_ was added as one of the sintering aids. Silicon nitride containing TiC released Ti ions and Y ions in SBF. The main reason for titanium alloy or TiO_2_ implants to damage cells is the wear of these two materials, which generates fine or nano particles, easily leading to infection at the transplantation site [61]. However, the self-lubricating property of silicon nitride reduces the production of material debris. In addition, previous studies have reported that commercial medical titanium alloy implants treated with fluoride and soaked in 35% hydrogen peroxide for 15 min released Ti ions concentrations of approximately 10 μg/L [62]. This is at the same level as the detected results. Therefore, the Ti ions release from silicon nitride is normal and it can be considered that Ti does not cause cytotoxicity. Y_2_O_3_ is recognized as a cytotoxic substance. In the preparation of silicon nitride, this compound plays a role in promoting the densification of silicon nitride. The impact of this substance on the cellular compatibility of silicon nitride needs to be considered. Through ICP measurement of the extract, the maximum Y ions concentration did not exceed 3 μg/L. In studies related to the cytotoxicity of Y_2_O_3_, the concentration of Y_2_O_3_ that causes toxicity to yeast cells is up to 20mg/5mL [63], 20 mg/L of Y_2_O_3_ concentration can inhibit plant rhizosphere growth [64], and the concentration of Y_2_O_3_ that causes toxicity to mouse osteoblasts is 40 μg/L [65]. These concentration levels are much higher than the Y ions concentration detected in the silicon nitride extract. At the same time, the cytotoxicity results have shown that cells can still proliferate normally when co-cultured with silicon nitride using Y_2_O_3_ as a sintering aid.

The sintering process of silicon nitride involves a liquid phase sintering process, which is mainly divided into particle rearrangement, dissolution–precipitation, and coarsening. During particle rearrangement, sintering additives such as TiC, Al_2_O_3_, and Y_2_O_3_ react with SiO_2_ on the surface of silicon nitride to form a eutectic liquid phase, which fills the gaps between particles. Under the influence of capillary force, the eutectic liquid phase flows, driving the particles to slide, deflect, and perform other behaviors [66,67]. This process expels enclosed pores inside the material and rapidly increases its densification. The dissolution of α-Si_3_N_4_ grains forms a supersaturated solution. The Si and N atoms in the liquid phase can migrate to other locations and precipitate to form nuclei, enabling the transformation from α-Si_3_N_4_ to β-Si_3_N_4_. During the growth of coarse grains, the silicon nitride ceramic becomes further densified. Ideally, the liquid phase evaporates entirely, and the Si_3_N_4_ crystals are tightly bonded in a directional manner. The grain boundaries exhibit pure phases and impurity-free zones, accompanied by significant macro-volume shrinkage [68]. As observed in Table 2, TiC-Si_3_N_4_ bioceramics with appropriate TiC addition ratios (3% and 5% wt) exhibit remarkable comprehensive performance advantages in terms of Vickers hardness, flexural toughness, compressive toughness, and fracture toughness. As He et al. suggested [69], gas pressure sintered (GPS) silicon nitride exhibits a flexural strength of 523 ± 55 MPa, while microwave sintered (MS) silicon nitride reaches 621 ± 63 MPa. Both values surpass that of alumina ceramics, which is 3–4 times higher than the flexural strength of PEEK. In terms of compressive strength, GPS silicon nitride achieves 2465 ± 210 MPa and MS silicon nitride 2586 ± 265 MPa, corresponding to approximately 2.5–3 times the compressive strength of titanium alloy and over 20 times that of PEEK. One method to enhance toughness of silicon nitride is the addition of a second-phase particle toughening agent. Adding TiC in appropriate amounts (3% and 5% wt) to silicon nitride falls into this category. The toughening mechanism involves the uniform distribution of TiC particles in the silicon nitride ceramic matrix. External pressure applied to silicon nitride may lead to the generation of microcracks. When cracks propagate to TiC due to external forces, the TiC acts as a pinning point, causing the cracks to deflect around it. This extends the crack propagation path and increases the energy consumed during fracture, thereby enhancing the fracture toughness of the silicon nitride ceramic [38,59]. However, excessive TiC addition can lead to excessive microcracks, which can easily connect, compromising the strength. Excessively high amounts of additives can result in a significant presence of residual liquid phase after the completion of phase transformation and densification processes. This residual liquid phase accumulates at the grain boundaries of Si_3_N_4_, negatively affecting the material. When the TiC content reaches between 8% and 13% wt, the mechanical properties of silicon nitride decrease significantly. Excessive TiC addition leads to its accumulation at the grain boundaries of Si_3_N_4_, significantly affecting the material’s density. Therefore, the mechanical properties of silicon nitride bioceramics decline significantly when the TiC content exceeds 8% wt.

Although the hardness of silicon nitride is significantly higher than the mechanical strength of human bones, not all biomedical materials are required to exhibit hardness values comparable to those of human bone. For certain load-bearing and wear-critical implant components—such as dental restorations, articulating joint components, and antibacterial structural implants—higher hardness is often necessary to enhance wear resistance, surface stability, and long-term mechanical reliability. Silicon nitride–based bioceramics are primarily designed for such applications rather than for direct load sharing with cancellous or cortical bone. Moreover, the high hardness of silicon nitride containing TiC composites contributes to improved wear resistance, which is critical for reducing debris generation and mitigating implant-induced inflammatory responses. For example, in total hip arthroplasty (THA), one of the most critical factors influencing clinical outcomes is the wear performance of the femoral head and acetabular components. Excessive wear particles, rather than material hardness itself, pose greater biological risks. Therefore, the increased hardness of the present materials may be beneficial for their long-term *in vivo* performance.

Using the CCK-8 Kit to assess the cytotoxicity of silicon nitride with different TiC addition amounts, it was demonstrated that the presence and increasing concentration of TiC did not affect the biocompatibility of silicon nitride. The cell viability of the control material is significantly weaker compared to that of silicon nitride containing TiC. Si and N are the main constituents of silicon nitride, and the rapid cell growth on silicon nitride could be attributed to these elements. Zeng et al. have reported that after 5 days of cultivation, the cell growth rate (indicated by OD values) on 3D printed Si_3_N_4_ material is significantly higher than that on the other three materials, indicating that among these four materials, 3D printed Si_3_N_4_ is more suitable for cell growth [70]. Kim et al. found that Si ions promote the proliferation and differentiation of osteoblasts as well as the expression of related markers [71]. Pezzotti et al. were the first to demonstrate that Si and N elements can simultaneously stimulate the differentiation of osteosarcoma and mesenchymal cells, as well as the activity of osteoblasts [17]. TiC is also commonly used as a mechanical performance enhancer for biomaterials, either as a coating or as an additive to enhance material properties. Yoshida et al. prepared TiC/Ti and TiC/Ti-15Mo ceramic metals for joints and measured good biocompatibility of both ceramics [72]. Balázsi et al. prepared TiC/a:C nano-thin films and cocultured them with MG63 cells, demonstrating their favorable biocompatibility. Ti and C are suitable elements for optimizing surface chemistry and promoting rapid bone integration [73]. Multiple studies have shown that implants coated with TiC or TiC films cocultured with cells enable good cell proliferation on the TiC coating or film, improving the material’s biocompatibility. Furthermore, research has demonstrated that TiC can stimulate the adhesion, proliferation, and activity of osteoblasts, inducing better bone–implant contact.

Cell adhesion represents the initial stage of the cellular response to biomaterials, and it has an impact on cell spreading and proliferation on the material surface [74]. Cell adhesion to materials belongs to focal adhesion, whose mechanism involves the chemical binding of cell pseudopods with protein molecules tightly adhered to the material surface. This is achieved through transmembrane integrins at the tips of pseudopods, which connect to actin in the cytoskeleton on one end and anchor to adsorbed proteins on the material surface through fibronectin on the other end. The expression of integrins is influenced by the surface properties of materials, such as surface roughness, microstructure, and wettability [75]. Both excessively smooth and excessively rough material surfaces are unsuitable for cell adhesion, whereas an appropriate surface roughness is beneficial for adhesion. Since different cell types possess distinct size characteristics, the optimal surface roughness for adhesion varies accordingly. For osteoblasts, a surface roughness of 200–700 nm is conducive to cell adhesion. Combined with the material surface morphology in Figure 1 and the surface roughness in Figure 3b, the surface morphology and roughness of silicon nitride with different TiC contents provide good adhesion sites for cells, resulting in good adhesion and the presence of more pseudopods.

Bacteria are prone to adhere to the surface of implants, colonize and form bacterial biofilms, often resulting in inflammatory reactions and implant failure [29]. Antibacterial properties are an important metric for bioceramics. The colony count results in Figure 7 initially indicate that the presence of TiC does not affect the antibacterial properties of silicon nitride. However, the CFU counts of all silicon nitride–based materials, including both TiC-containing and TiC-free samples, were significantly lower than those of the control materials (alumina, titanium alloy, and polyether ether ketone). This finding demonstrates that the antibacterial activity primarily originates from the silicon nitride matrix itself rather than from the TiC additive. The absence of significant differences among the silicon nitride containing TiC samples should not be regarded as a limitation; instead, it reflects the stability of their antibacterial performance. This result indicates that the incorporation of TiC does not compromise the intrinsic antibacterial function of silicon nitride, which is highly advantageous for biomedical applications, as it allows for the enhancement of mechanical properties without sacrificing antibacterial efficacy. The surface morphology and chemical environment of materials can also affect bacterial adhesion [26]. Surface roughness is one of the factors that influence the antibacterial performance of materials. It is generally believed that smoother surfaces exhibit lower bacterial adhesion, but Whitehead and Verran disputed this based on experimental results, and subsequent studies have supported this contention. The relationship between roughness and bacterial adhesion is non-linear. When surface features approximate the size or spacing of bacteria on the micrometer scale, it seems to enhance bacterial adhesion [76]. Wettability can also affect bacterial adhesion on materials, where hydrophilic surfaces inhibit the adhesion of hydrophobic bacteria. Silicon nitride can react with water in an aqueous environment to produce Si-OH groups on the surface. The hydrolysis reaction is as follows [77]:

Si_3_N_4_ + 6H_2_O → 3SiO_2_ + 4NH_3_ (1)

The ammonia produced dissolves to form NH_4_^+^ and OH^−^, raising the local pH. The silica formed further reacts with water to produce surface hydroxyl groups [Si–OH]. In the presence of electrolytes such as NaCl, ion exchange occurs:

Si–OH + Na^+^ → Si–O^−^Na^+^ + H^+^ (2)

This reaction will leave the surface negatively charged. The released H^+^ is partially neutralized by the generated NH_3_, contributing to a relatively stable or slightly alkaline pH in the system.

The negatively charged surface can thus produce electrostatic repulsion against bacteria with a net negative charge on their surface, thereby inhibiting bacterial adhesion to the silicon nitride surface [78]. In addition to the above factors, the chemical mechanism of silicon nitride’s resistance to bacteria has also been elucidated. There are differences in the resistance mechanisms of silicon nitride against Gram-positive and Gram-negative bacteria [79,80,81,82]. For Gram-positive bacteria, reactive nitrogen species (RNS) are formed on the surface of silicon nitride, which can disrupt the DNA/RNA of Gram-positive bacteria. Conversely, for Gram-negative bacteria, silicon nitride promotes the degradation of nucleic acids and nucleotide-containing molecules in bacteria to form uric acid, leading to the accumulation of NH_3_ and NH_4_^+^ in bacteria and osmotic stress, ultimately resulting in bacterial lysis. The NH_3_ or NH_4_^+^ dissolved from silicon nitride can cause an increase in local environmental pH, which is considered unfavorable for bacterial adhesion [26,79]. In this study, fewer colonies of *S. aureus* and *E. coli* were observed on the agar plates corresponding to silicon nitride compared to the control materials. Based on the proposed antibacterial theory of silicon nitride and the factors that affect bacterial adhesion, the cause of this phenomenon may be the combined effect of biochemical reactions occurring on the surface of silicon nitride in an aqueous environment and the properties of the material surface, but further research is warranted to elucidate this relationship.

The silicon nitride containing TiC can be used in biomedical applications that require load bearing and wear resistance, such as dental restoration, joint surface components, spinal implants, and antibacterial structural implants. Owing to their high density, excellent mechanical strength, high hardness, and antibacterial activity, these materials are particularly suitable for permanent or long-term implant components that require structural stability and resistance to mechanical degradation in physiological environments. The intrinsic antibacterial properties of silicon nitride contribute to reducing the risk of implant-associated infections, while its high hardness and superior wear resistance help minimize debris generation and mitigate implant-induced inflammatory responses. In addition, silicon nitride exhibits radiolucency and excellent imaging clarity, producing minimal imaging artifacts. This allows clinicians to clearly visualize the underlying bone and surrounding soft tissues, providing significant advantages for postoperative monitoring and clinical assessment.

## 5. Conclusions

This study systematically investigated the effects of varying TiC contents on silicon nitride, with particular emphasis on identifying an optimal TiC concentration that achieves a balance among mechanical performance, cytocompatibility, and antibacterial behavior. Silicon nitride containing 5% wt TiC exhibits significant enhancement in fracture toughness. Silicon nitride containing TiC does not release harmful metal ions in solution. Silicon nitride containing TiC has no cytotoxicity. MC3T3-E1 cells can adhere normally to silicon nitride with different amounts of TiC addition, while maintaining normal cytoskeleton morphology and intercellular connections. Silicon nitride containing TiC also exhibits significant antibacterial activity against Staphylococcus aureus and Escherichia coli. While most previous studies have primarily focused on mechanical properties, the present work provides a comprehensive correlation between microstructure, mechanical performance, and biological responses within the silicon nitride material system. However, further in-depth research is needed on the osteogenic properties of silicon nitride containing TiC and its impact on animal physiological activities. Future work will focus on combining *in vitro* and *in vivo* experiments with clinical trial data to improve the preparation process of silicon nitride and manufacture high-performance silicon nitride containing TiC implants.

## Figures and Tables

**Table 1:** EDS analysis of surface elements of silicon nitrides containing TiC.

Materials	C (cps)	N (cps)	O (cps)	Mg (cps)	Al (cps)	Si (cps)	Ti (cps)
SN + 3% TiC	1.51 k	12.77 k	4.69 k	3.74 k	5.26 k	125.80 k	0.44 k
SN + 5% TiC	1.89 k	13.16 k	3.90 k	3.84 k	5.41 k	123.58 k	1.66 k
SN + 8% TiC	1.94 k	13.48 k	3.73 k	3.78 k	5.22 k	121.78 k	2.38 k
SN + 13% TiC	1.98 k	13.75 k	3.68 k	3.55 k	5.07 k	119.68 k	3.45 k

**Table 2:** Mechanical properties of silicon nitrides and control materials.

Materials	Vickers Hardness (GPa)	Flexural Strength (MPa)	Compressive Strength (MPa)	Fracture Toughness (MPa·m^1/2^)
SN	14.73 ± 0.3	523 ± 31.9	2465 ± 131.4	7.47 ± 0.583
SN + 3% TiC	15.02 ± 0.28	571 ± 29.3	2537 ± 132.9	8.35 ± 0.622
SN + 5% TiC	15.23 ± 0.33	532 ± 30.9	2431 ± 133.9	8.53 ± 0.663
SN + 8% TiC	14.51 ± 0.31	482 ± 32.7	2315 ± 138.4	7.66 ± 0.642
SN + 13% TiC	14.13 ± 0.39	435 ± 31.0	2114 ± 131.9	6.55 ± 0.601
Alumina [58]	14.00–16.00	300–500	2000–3000	4–5
Titanium alloy [58]	3.40	/	950–990	75
PEEK [58]	/	160–180	130–140	/

## Data Availability

The raw data supporting the conclusions of this article will be made available by the authors on request.
